# Silencing ATF3 Might Delay TBHP-Induced Intervertebral Disc Degeneration by Repressing NPC Ferroptosis, Apoptosis, and ECM Degradation

**DOI:** 10.1155/2022/4235126

**Published:** 2022-04-15

**Authors:** Yongjin Li, Dayu Pan, Xuke Wang, Zhenxin Huo, Xiaojing Wu, Jianhua Li, Jiasong Cao, Haiwei Xu, Lilong Du, Baoshan Xu

**Affiliations:** ^1^Department of Minimally Invasive Spine Surgery, Tianjin Hospital, No. 406, Jiefangnan Road, Hexi District, Tianjin, China 300211; ^2^Graduate School, Tianjin Medical University, 22 Qixiangtai Road, Tianjin, China; ^3^Department of Minimally Invasive Spine Surgery, Luoyang Orthopedic-Traumatological Hospital, Qiming Road, Luoyang City, China 471002; ^4^Department of Surgery Critical Care Medicine, Beijing Shijitan Hospital, Capital Medical University, No. 10 Tieyi Road, Haidian District, Beijing 100038, China; ^5^Department of Orthopaedics, Tianjin Haihe Hospital, Tianjin 300350, China; ^6^Tianjin Key Lab of Human Development and Reproductive Regulation, Tianjin Central Hospital of Obstetrics and Gynecology, Nankai University, Tianjin 300199, China

## Abstract

Intervertebral disc degeneration (IDD), being the predominant root cause of lower back pain, has led to an enormous socioeconomic burden in the world. Ferroptosis is an iron-dependent nonapoptotic and nonpyroptotic programmed cell death associated with an increase in reactive oxygen species (ROS), which has been implicated in the pathogenesis of IDD. Activation transcription factor 3 (ATF3) is widely reported to promote ferroptosis and apoptosis in multiple diseases, but its roles and underlying regulatory mechanism in IDD have not been identified. FAoptosis is defined as a mixed cell death consisting of ferroptosis and apoptosis. The loss- and gain-of-function experiments demonstrated that ATF3 positively regulated *tert*-butyl hydroperoxide- (TBHP-) induced nucleus pulposus cell (NPC) FAoptosis, ROS production, inflammatory response, and extracellular matrix (ECM) degradation. Furthermore, silencing ATF3 ameliorated the progression of IDD *in vivo*, whereas its overexpression showed the opposite phenotype. Bioinformatics analysis and molecular experiments corroborated that ATF3 is a direct target of miR-874-3p, suggesting that the upregulation of ATF3 in IDD might be caused at least in part due to the downregulation of miR-874-3p in IDD, thereby relieving the inhibition of ATF3 by miR-874-3p. The findings revealed that ATF3 has the potential to be used as a promising therapeutic target against IDD.

## 1. Introduction

Increasing epidemiological data showed that lower back pain (LBP) is one of the most prevalent symptoms with a higher disability rate, contributing to enormous economic losses worldwide [1–3]. Intervertebral disc degeneration (IDD) is the predominant root cause that triggers LBP [4]. The degradation of the nucleus pulposus (NP), which is located at the center of the intervertebral disc (IVD) with the annulus fibrosus (AF) around it, is an important initiation factor for IDD. NP cell (NPC) degeneration could trigger a series of pathological events, such as the imbalance between the extracellular matrix (ECM) synthesis and catabolism, the predominant dysregulated synthesis of Aggrecan and collagen type II (COL2A1) [4–6], the abnormal increase in NPC death (ferroptosis, apoptosis, and pyroptosis, etc.) [5–9], proinflammatory cytokine (including IL-6, TNF-*α*, and IL-1*β*, etc.) secretion [4, 6, 10], and oxidative stress [8, 9]. TNF-*α* and IL-1*β* are the two most commonly studied cytokines in IDD. These pathological events further aggravate the progression of IDD and trigger LBP. Therefore, maintaining the balance in the microenvironment for the survival of NPCs and their normal physiological functioning is of great significance for alleviating or delaying IDD and reducing socioeconomic burden.

Ferroptosis, an iron-dependent nonapoptotic and nonpyroptotic programmed cell death, is characterized by an iron-dependent form of the increase of lipid peroxidation and reactive oxygen species (ROS) and changes in the expression of key genes [11]. Several studies have reported that impaired ferroptosis is involved in the pathogenesis of IDD [7–10]. Emerging evidence proved that the repression of ferroptosis is an effective strategy to alleviate the progression of IDD [8, 10]. Cystine transporter solute carrier family 7 member 11 (SLC7A11) is a ferroptosis marker, which could promote cystine uptake and suppress ferroptosis [12–15]. Activation transcription factor 3 (ATF3) was corroborated to enhance ferroptosis by directly binding to the *SLC7A11* promoter and inhibiting *SLC7A11* expression in erastin-induced HT1080 cells [13]. As a hub gene of the cellular adaptive response network, ATF3 is widely involved in the regulation of cell metabolism and intracellular signaling pathways under normal physiological conditions [16]. Thus, we speculated the involvement of mixed cell death (FAoptosis: consisting of ferroptosis and apoptosis), which is mediated by ATF3. Furthermore, previous studies have shown that ATF3 expression is significantly increased in the degenerative NP tissues of rats [17, 18]. However, the precise role of ATF3 in the progression of IDD and the underlying regulatory mechanism has not been identified.

MicroRNA (miRNA) expression is temporal, and dysregulation in their expression is a hallmark pathological feature of IDD [19]. Accumulating evidence from animal experiments has demonstrated that miRNAs have therapeutic potential in IDD diseases [20–22]. miRNAs are short noncoding RNAs (ncRNAs), approximately 21-24 nucleotides long, which negatively modulate target gene expression and its downstream signaling pathway by directly binding to their target genes' 3′-UTR (untranslated regions), thereby mediating NPC growth and apoptosis, inflammatory response, ECM metabolism, and oxidative stress [19–22]. Thus, we speculated that miRNA is likely to be an upstream regulator of ATF3. The current study intended to identify the novel functional miRNA that directly binds to ATF3 in IDD.

In our study, the ferroptosis-related genes were listed using the FerrDb database (http://www.zhounan.org/ferrdb/) [23], and the human lumbar NP tissue-related microarray dataset (miRNA: GSE116726) was obtained from the public open Gene Expression Omnibus (GEO) database (http://www.ncbi.nlm.nih.gov/geo) [24]. Bioinformatics analysis was performed to identify key molecules. We found that ATF3 is a key transcription factor (TF) and significantly upregulated in IDD. Moreover, ATF3 has sequences complementary to the miR-874-3p seed region. Subsequently, we investigated the role and underlying mechanisms of ATF3 in IDD by performing *in vitro* and *in vivo* experiments.

## 2. Online Methods

### 2.1. Ethics Statement and NP Tissue Collection

The study was supervised and approved by the Tianjin Hospital Ethics Committee. All donors signed the informed consent form before surgery. We collected human degenerative NP tissues from 30 patients with IDD who were diagnosed with lumbar disc herniation, lumbar spinal stenosis, and lumbar spondylolisthesis and then subjected to lumbar nucleotomy. The normal tissues were obtained from 16 patients with scoliosis, fresh thoracolumbar fracture, and spinal cord injury who were undergoing surgery. Patients with rheumatoid arthritis, immune diseases, seropositive and negative spondyloarthropathy, thyroid diseases, tumors, and tuberculosis were excluded from the study. Pfirrmann's classification method was utilized to assess the severity of IDD [25]; patients with Pfirrmann grades I-II were assigned to the normal group, whereas those with Pfirrmann grades III-IV constituted the mild degeneration group and grade V constituted the severe degeneration group. The detailed data for each patient with different diseases are shown in Supplementary Table 1.

### 2.2. TF Enrichment Analysis (TFEA)

The ferroptosis-related genes were downloaded from the FerrDb database (http://www.zhounan.org/ferrdb/) [23], including ferroptosis driver, suppressor, marker, and multirole regulator, which are shown in Supplementary Table 2. ChIP-X Enrichment Analysis 3 (ChEA3) (https://maayanlab.cloud/chea3/#top) is a TF enrichment analysis (TFEA) tool that ranks the TFs associated with genes [26]. We used ChEA3 to explore TF targets and to annotate the main biological functions of the genes involved in the ferroptosis. The integrated MeanRank TF Ranks were analyzed through the interaction of the given lists of genes and previously annotated TF targets assembled from multiple resources, encompassing GTEx coexpression, ARCHS4 coexpression, ENCODE ChIP-seq, gene sets from individual ChIP-seq publications, ReMap ChIP-seq, and Enrichr Queries.

### 2.3. Analysis of miRNA Microarray Dataset

R software limma package [27] was used to analyze the miRNA (GSE116726) microarray dataset. The screening criteria for differentially expressed miRNAs (DEMs) were as follows: -log_10_ adjusted *P* value (adj.P) >2 and |log_2_*fold* − *change*(*FC*)| > log_2_6. The volcano plots and clustering heat map were outputted using R software.

### 2.4. Bioinformatics Analysis

The potential upstream miRNAs of ATF3 were predicted using TargetScan (http://www. http://targetscan.org/vert_72/) [28], mirDIP (http://ophid.utoronto.ca/mirDIP/) [29], miRecords (http://miRecords.umn.edu/miRecords) [30], and miRWalk (http://mirwal k.umm.uni-heidelberg.de/faqs/) [31] databases as well as the GSE116726 dataset. Conversely, the target genes of key miRNA were predicted by miRanda (http://www.microrna.org/microrna/home.do) [32], mirDIP [29], miRecords [30], and miRWalk [31] databases as well as ferroptosis genes.

### 2.5. Human NPC Culture and Treatment

The precise method was described in our previously study [33, 34]. Human primary NPCs were purchased from ScienCell Research Laboratories (ScienCell, Cat. #4800, USA). The cells were cultured in Nucleus Pulposus Cell Medium (NPCM, Cat. #4801, ScienCell, USA) supplemented with 10 mL fetal bovine serum, 5 mL NPC growth supplement, and 5 mL penicillin/streptomycin solution and maintained at 37°C and 5% CO_2_ in a humid environment. The medium was changed every 2 days. The NPCs were passaged once a week, and the well-grown NPCs were taken for subsequent experiments.

To simulate an oxidative stress microenvironment *in vitro*, the NPCs were treated with increasing concentrations (0, 10, 25, and 50 *μ*M) of *tert*-butyl hydroperoxide (TBHP) (Sigma, MO, USA) for 24 h, as described previously [8, 35–38].

### 2.6. Vector Construction and NPC Transfection

The ATF3 overexpression vector (pcDNA3.1+ATF3), ATF3 small interfering RNA (siRNA), miR-874-3p mimic, miR-874-3p inhibitor, and their corresponding negative control (NC) were obtained from JIAMAY BIOLAB (Beijing, China). The effects of silencing and overexpression were measured using qRT-PCR. Lipofectamine 8000 (Beyotime, China) was used to transfect plasmids or miR-874-3p or NCs into NPCs following the manufacturer's protocol. After 48 h transfection, NPCs were used to conduct the subsequent experiments.

### 2.7. RNA Extraction and Quantitative Real-Time RT-PCR

The TRlzol Reagent (Life Technologies, Thermo Fisher Scientific, USA) was used to extract total RNAs from NPCs or NP tissues. We used 1 *μ*g total RNAs and 1 *μ*L Geneseed® Enzyme Mix (Geneseed, Guangzhou, China) to reverse into 20 *μ*L complementary DNA (cDNA) using Geneseed® II First Strand cDNA Synthesis Kit (Geneseed, Guangzhou, China). Subsequently, 10 *μ*L Geneseed® qPCR SYBR® Green Master Mix (Geneseed, Guangzhou, China), 0.5 *μ*L forward primer, and 0.5 *μ*L reverse primer were prepared to perform qRT-PCR on the ABI 7500 system (Applied Biosystems, USA). The primers used in this study are the following: ATF3: forward 5′-GTGCCGAAACAAGAAGAAGG-3′, reverse 5′-TCTGAGCCTTCAGTTCAGC A-3′; miR-874-3p: forward 5′-ATGGTTCGTGGGCTGCCCTGGC-3′, Com reverse GTGCAGGGTCCGAGGT, reverse transcription GTCGTATCCAGTGCAGGGTC CGAGGTATTCGCACTGGATACGACCTCGGTCCC; U6: forward 5′-CTCGC TTCGGCAGCACA-3′, Com reverse AACGCTTCACGAATTTGCGT, reverse transcription: GTCGTATCCAGTGCAGGGTCCGAGGTATTCGCACTGGATACGA CCAAATATGGAAC; GAPDH: forward 5′-AGAAGGCTGGGGCTCATTTG-3′, reverse 5′-GCAGGAGGCATTGCTGATGAT-3′; Aggrecan: forward 5′-TGTAACC CAGGCTCCAAC-3′, reverse 5′-GCAGCCCACTTAGGTCC-3′; COL2A1: forward 5′-GCCTGAAGGGACACCG-3′, reverse 5′-CCAGGGATTCCATTAGCAC-3′. ATF3, Aggrecan, and COL2A1 expressions were normalized to GAPDH, whereas the miR-874-3p expression was normalized to U6. The relative expression levels of miR-874-3p, ATF3, Aggrecan, and COL2A1 were detected and analyzed using the 2^-△△^Ct method, as described previously [39].

### 2.8. Protein Extraction and Western Blot

The RIPA lysis buffer containing phenylmethanesulfonyl fluoride (Beyotime, Shanghai, China) was employed to extract proteins from NPCs or NP tissues. We then employed the Micro Bicinchoninic Acid Protein Assay kit (Beyotime, Shanghai, China) to evaluate the protein concentration. Next, the proteins were isolated through SDS-PAGE and then were transferred to polyvinylidene difluoride (PVDF) membranes (Millipore, Germany) at 350 mA for 90 min. Subsequently, the PVDF membranes were closed with 5% nonfat milk for 60 min and incubated overnight with primary antibody at 4°C and washed 10 min 3 times using phosphate-buffered saline with Tween-20. The primary antibodies include Aggrecan (Abcam, ab3778, 1 : 200), COL2A1 (Abcam, ab188570, 1 : 5000), SLC7A11 (Abcam, ab175186, 1 : 10000), c-CASP3 (Abcam, ab32351, 1 : 5000), IL-1*β* (Abcam, ab9722, 1 : 1000), SOD2 (Abcam, ab68155, 1 : 1000), and *β*-actin (Proteintech, 66009-1-1 g, 1 : 10000). Then, the membranes were incubated with a secondary antibody for 60 min at room temperature followed by washing again. Finally, the PVDF membranes were put into a chemiluminescent substrate and developed for 1-2 minutes. Then, the chemiluminescence system (Bio-Rad, CA, USA) was employed to detect the signals.

### 2.9. Flow Cytometry (FCM)

The Annexin V-FITC apoptosis detection kit (keyGEN, Jiangsu, China) was used to assess NPC apoptosis rates. Annexin V-FITC was matched with propidium iodide (PI) to distinguish NPCs in different stages of apoptosis. The NPCs were stained with 1.25 *μ*L Annexin V-FITC and 10 *μ*L PI. Then, the FlowJo VX10 software was used to cope with the data. On the scatter plot of the bivariate FCM, Q2 represented the late apoptotic and necrotic NPCs, whereas Q3 represented the early apoptotic NPCs, so Q2+Q3 reflected the apoptosis rate of NPCs.

### 2.10. Enzyme-Linked Immunosorbent Assay (ELISA)

Human IL-1*β* ELISA kits (Elabscience, E-EL-H0149c) were used to measure the concentration of IL-1*β* in NPCs in different treatment conditions. The IL-1*β* antibody was added to the ELISA well, and standards and samples were added to the microplates. Each standard and sample were detected by ELISA at a wavelength of 450 nm. IL-1*β* concentration was calculated according to the absorbance value.

### 2.11. Dual-Luciferase Reporter Assay

The putative binding sites of miR-874-3p with ATF3 mRNA 3′-UTR were predicted using the TargetScan database [28]. The luciferase reporter vectors (psiCHECK2-Firefly luciferase-Renilla luciferase containing ATF3 wild-type (WT) sequence or mutant (MUT) sequence) were obtained from Guangzhou Geneseed Biotech Co. (Guangzhou, China). Human embryonic kidney (HEK) 293T cells were added to 24-well plates at a density of 1 × 10^5^ cells per well. Subsequently, 1 *μ*g vectors and 100 *μ*L miR-874-3p mimic or mimic NC were cotransfected to HEK-293T cells using 2 *μ*L Lipofectamine 8000 (Beyotime, China). The Luciferase Assay Kit (Promega, Madison, WI, USA) was used to measure the luciferase activity of WT ATF3 or MUT ATF3. The reporter genes' activation degree was calculated between different samples according to the obtained ratio of the relative light unit (RLU) value detected by the Renilla luciferase is divided by the RLU value detected by the Firefly luciferase.

### 2.12. Construction and Treatment of Rat IDD Model

Forty adult Sprague–Dawley (SD) rats (3 months) were obtained from the American Charles River Laboratories to construct a rat IDD model, using a needle puncture method previously described by Ji et al. [21]. They were randomly divided into five groups, namely, control, siRNA NC, ATF3 siRNA (ATF3 si), vector NC, and ATF3 overexpression vector (ATF3), of which thirty-two rats underwent surgery and the remaining eight rats without any intervention as NCs. The rats were anesthetized through intraperitoneal injection of 90 mg/kg ketamine and 10 mg/kg xylazine. Under the guidance of fluoroscopy, a 31 G needle was utilized to puncture the tail Co8/9 from the dorsal side and passed through the annulus fibrosus, inserted into the NP region about 1.5 mm, and then rotated 180° in the axial direction and held for 10 s. The Co7/8 and Co9/10 were left undisturbed as a control. Four weeks and eight weeks after the surgery, 2 *μ*L adenoviral containing ATF3 overexpression vector or ATF3 siRNA or corresponding NCs were injected into the ATF3 group or ATF3 si group or siRNA NC and vector NC group using a 31 G needle, respectively.

### 2.13. MRI Examination

MRI examinations were conducted using a 1.5 T animal-specific MRI system to obtain T2-weighted images (repletion time: 3000 ms; echo time: 80 ms; field of view: 200 mm × 200 mm; thickness: 1.4 mm) in the midsagittal plane at 12 weeks after the surgery. The severity degree of IDD was evaluated via MRI based on Pfirrmann's classification method [25]. The Pfirrmann grade is divided into five grades, ranging from grade I for normal IVD to grade V for severely degenerative IVD. More specifically, grade I: the IVD is of normal height and homogeneous structure with a bright hyperintense white signal intensity; grade II: the IVD is of normal height and inhomogeneous structure with a hyperintense white signal. The border between NP and AF is distinct with or without horizontal gray bands. Grade III: the IVD is of normal or slightly decreased height and inhomogeneous structure with an intermediate gray signal intensity. The border between NP and AF is indistinct. Grade IV: the IVD is of normal or moderately decreased height and inhomogeneous structure with a hypointense dark gray signal intensity. The border between NP and AF disappeared. Grade V: there is an inhomogeneous IVD structure with a hypointense black signal intensity. The border between NP and AF disappeared, and the IVD space collapsed [25]. The average score of the punctured IVDs was calculated as the degeneration grade of each rat.

### 2.14. Histological Assessment and Immunohistochemistry Staining

The rats were killed via overdose of an intraperitoneal injection of pentobarbital sodium, and the tail NP tissues were collected. The NP tissues were fixed in 4% paraformaldehyde for 48 h, decalcified in ethylenediaminetetraacetic acid (EDTA), and embedded in paraffin. Then, the tissues were cut into 5 *μ*m thick sections along the midsagittal plane for staining. For Hematoxylin and Eosin (H&E) and Safranin O-fast green (SO) staining, the histological scores ranged from 0 to 15 points according to five categories [21]. The higher the score, the more serious the degree of intervertebral disc degeneration. The five categories were described as follows: (1) the NP morphology: score 0: round shape and the NP constitutes beyond 75% of the IVD region; score 1: round shape and the NP constitutes 50-75% of the IVD region; score 2: round shape and the NP constitutes 25-50% of the IVD region; and score 3: round shape and the NP constitutes less than 25% of the IVD region. (2) The AF morphology: score 0: intact collagen lamellae with no ruptures; score 1: inward bulging, ruptured, or serpentine fibers constitute less than 25% of the AF; score 2: inward bulging, ruptured, or serpentine fibers constitute 25−50% of the AF; and score 3: inward bulging, ruptured, or serpentine fibers constitute beyond 50% of the AF. (3) The NP cellularity: score 0: stellar-shaped cells with a proteoglycan matrix located at the periphery, evenly distributed; score 1: more stellar cells than round cells; score 2: mostly large, round-shaped cells, separated by dense areas of proteoglycan matrix; and score 3: large, round cells, separated by dense areas of proteoglycan matrix. (4) The AF cellularity: score 0: fibroblasts constitute beyond 90% of the cells; score 1: fibroblasts constitute 75-90% of the cells; score 2: intermediate; and score 3: chondrocytes constitute beyond 75% of the cells. (5) The boundary between the NP and AF: score 0: normal, without any interruption; score 1: minimal interruption; score 2: moderate interruption; and score 3: severe interruption [21]. For immunohistochemical analysis, the paraffin-embedded sections were deparaffinized, rehydrated, and incubated in EDTA to retrieve the antigen. Then, the sections were incubated with the primary antibodies overnight at 4°C, followed by incubation with a secondary antibody for 60 min at room temperature. The nuclei were stained with DAPI. All the pictures were photographed using the BX53 microscope (Olympus, Tokyo, Japan).

### 2.15. Statistical Analysis

The experiments were performed at least three times. All data were analyzed and outputted as figures using the GraphPad Prism software version 6. The statistical significance between the two groups was compared using the unpaired Student's *t-*test, whereas the differences among more than two groups were assessed using one-way ANOVA followed by Tukey's multiple comparison test. Results are presented as mean ± standard deviation. The *P* values < 0.05 were deemed to represent statistical significance. ∗ represents *P* < 0.05, ∗∗ represents *P* < 0.01, and ∗∗∗ represents *P* < 0.001.

## 3. Results

### 3.1. ATF3 Was Significantly Upregulated in IDD Patients and TBHP-Treated NPCs

**Figure 1:**
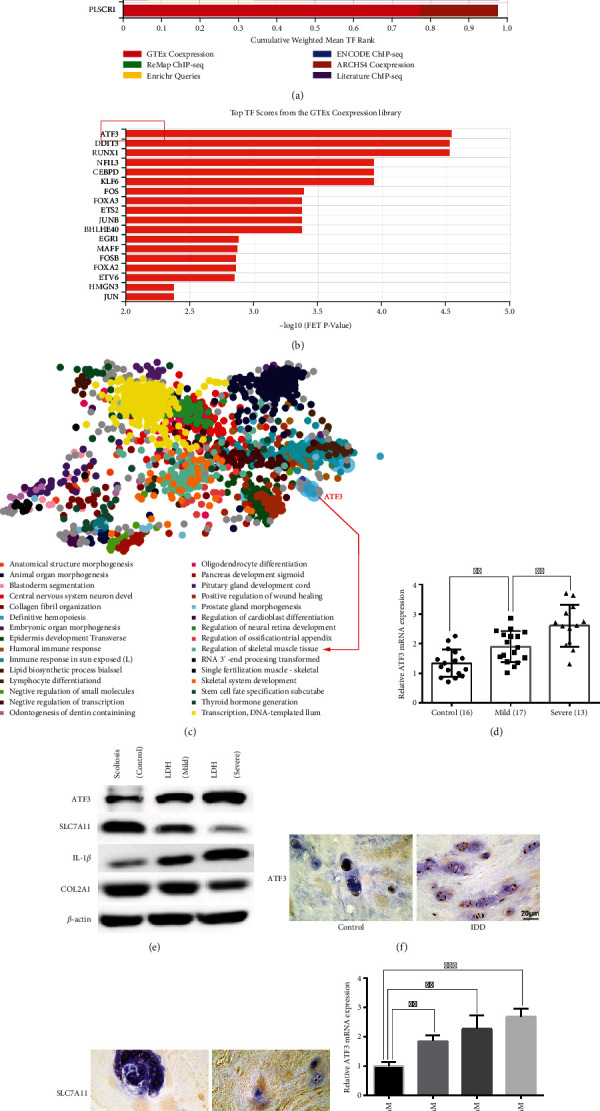
ATF3 was significantly upregulated in IDD patients and TBHP-treated NPCs. (a) The weighted library contribution to integrated MeanRank TF Ranks. (b) Top TF scores from the GTEx coexpression library. (c) GO enrichment analysis of the ferroptosis genes. (d) The mRNA expression level of ATF3 in normal NP tissues (*n* = 16), mild degenerative tissues (*n* = 17), and severe degenerative NP tissues (*n* = 13) was measured by qRT-PCR. ^∗∗^*P* < 0.01. (e) The protein expression levels of ATF3, SLC7A11, IL-1*β*, and COL2A1 in normal and degenerative NP tissues were measured by western blotting. (f, g) Immunohistochemical analysis of the expression level of ATF3 and SLC7A11 in human degenerative NP tissues. Scale bar = 20 *μ*m. (h) The mRNA expression level of ATF3 in NPCs treated with different doses of TBHP for 24 hours was measured by qRT-PCR. ^∗∗^*P* < 0.01, ^∗∗∗^*P* < 0.001.

Lu et al. [8] observed, using transmission electron microscopy, that TBHP-treated human NPCs showed smaller mitochondria with increased membrane density, a typical morphological feature of ferroptosis. Yang et al. [9] confirmed that the expression level of the typical ferroptosis marker GPX4 was downregulated in the human degenerative NP tissues. These reports implied a role of ferroptosis in the degeneration of NPCs and tissues. However, the pathology of ferroptosis in IDD remains unclear. Specific TFs play critical roles in mediating gene expression, signal transduction, and cell death. To gain insight into the key TFs that may regulate ferroptosis, 248 ferroptosis-related genes were listed using the FerrDb database and mapped into the ChEA3 website to perform TFEA. The results suggested that ATF3 ranked second in the weighted library contribution to integrated MeanRank TF Ranks and ranked first in the GTEx coexpression library (Figures 1(a) and 1(b)). GO enrichment analysis of ferroptosis-related genes suggested that ATF3 is involved in the regulation of skeletal muscle tissue development (Figure 1(c)). Consistent with the previous findings [17, 18], our results showed that the mRNA and protein expression levels of ATF3 were high in mildly degenerated NP tissues and higher in severely degenerated NP tissues compared with the relatively normal NP tissues (Figures 1(d) and 1(e)). Le Maitre et al. [10] showed that the expression of IL-1*β* and its receptor IL-1 RI was significantly higher than that of TNF-*α* and its receptor TNF RI in IDD, indicating that IL-1*β* may have a more important role than TNF-*α*. We detected the expression levels of several key proteins in IDD. We observed that ATF3 and IL-1*β* levels were positively correlated with the severity of IDD, whereas COL2A1 and SLC7A11 levels were negatively correlated with the severity of IDD (Figure 1(e)). Furthermore, immunohistochemical analysis unveiled that the expression level of ATF3 was higher, and SLC7A11 was lower in human degenerative NP tissues (Figures 1(f) and 1(g)). Thus, ATF3 was considered one of the key ferroptosis-related genes in IDD.

TBHP has higher stability and slower release than hydrogen peroxide [40], which is widely reported to simulate an oxidative microenvironment *in vitro* [8, 35–38]. We observed that TBHP could increase the mRNA expression levels of ATF3 in a dose-dependent manner in NPCs (Figure 1(h)). Furthermore, Kang et al. [38] observed that NPC viability was more than 50% even after treatment with 50 *μ*M TBHP. Thus, we chose 50 *μ*M of TBHP as the optimum concentration to stimulate NPCs in subsequent experiments. Taken together, these results suggested that ATF3 was significantly upregulated in IDD patients and TBHP-treated NPCs.

### 3.2. Identification of NPC Transfection Ability

**Figure 2:**
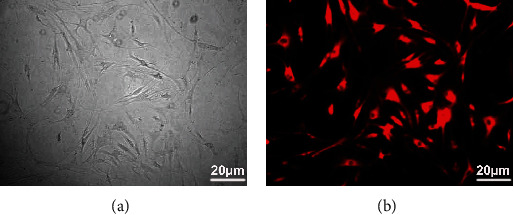
Identification of NPC transfection ability. The small interfering RNA NC was transfected into the NPCs to verify the transfection ability of NPCs using Lipofectamine 8000. Scale bar = 20 *μ*m. (a) Before transfection; (b) after transfection.

The NPCs were purchased from ScienCell Research Laboratories and were previously demonstrated to be the true NPCs. To prove whether the NPCs are transfectable, we transfected the CY3-labeled siRNA NC into NPCs to evaluate transfection efficiency. We observed red fluorescence in posttransfected NPCs compared to pretransfected NPCs (Figures 2(a) and 2(b)), suggesting that the siRNA NC can be successfully transfected into NPCs. These findings confirmed that the NPCs had a certain transfection ability.

### 3.3. The Role of ATF3 in FAoptosis, ECM Degradation, ROS Production, and IL-1*β* Secretion in NPCs

**Figure 3:**
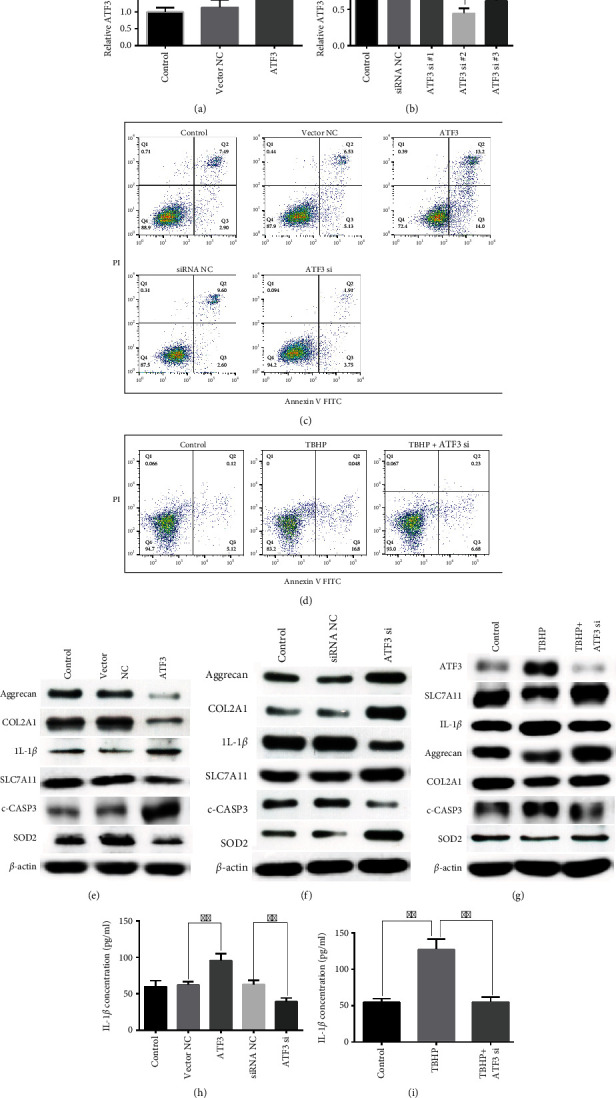
Roles of ATF3 in the FAoptosis, ECM degradation, ROS production, and IL-1*β* secretion in NPCs. (a, b) The expression level of ATF3 in NPCs was measured by qRT-PCR after transfection with ATF3 overexpression vector, ATF3 si, or corresponding NC. ^∗^*P* < 0.05, ^∗∗^*P* < 0.01, and ^∗∗∗^*P* < 0.001. (c) The effect of ATF3 or ATF3 si on NPC apoptosis was measured using flow cytometry assay. Representative dot plots of apoptosis were shown after Annexin V-FITC/PI dual staining. ^∗∗^*P* < 0.01 and ^∗∗∗^*P* < 0.001. (d) The NPC apoptosis was measured using flow cytometry assay after being treated with TBHP or TBHP+ATF3 si. ^∗∗∗^*P* < 0.001. The protein expression levels of ATF3, SLC7A11, IL-1*β*, Aggrecan, COL2A1, c-CASP3, and SOD2 in ATF3-overexpressing NPCs (e) or ATF3 knockdown NPCs (f) were measured by western blot assay. (g) The protein expression levels of ATF3, SLC7A11, IL-1*β*, Aggrecan, COL2A1, c-CASP3, and SOD2 in NPCs were measured using western blot assay after being treated with TBHP or TBHP+ATF3 si. (h) ELISA was used to measure the concentration of IL-1*β* in NPCs after transfection with ATF3 overexpression vector, ATF3 si, or corresponding NC. ^∗∗^*P* < 0.01. (i) The concentration of IL-1*β* in NPCs was measured using ELISA after being treated with TBHP or TBHP+ATF3 si. ^∗∗^*P* < 0.01.

To further elucidate the role of ATF3 in the FAoptosis, ECM degradation, ROS production, and IL-1*β* secretion in NPCs, we conducted loss- and gain-of-function experiments. First, we transfected the ATF3 overexpression vector (ATF3) or ATF3 si into NPCs to construct NPCs overexpressing ATF3 or ATF3-knockouts, respectively. The results revealed that ATF3 significantly elevated and ATF3 si significantly repressed the expression of ATF3 in NPCs (Figures 3(a) and 3(b)). Given that cleaved caspase3 (c-CASP3) is the executor of cell apoptosis and superoxide dismutase 2 (SOD2) is an antioxidant protein, we measured their levels to reflect the NPC apoptosis and the level of oxidative stress in the microenvironment of the intervertebral disc, respectively. The NPC apoptosis rate, ECM degradation, and IL-1*β* and c-CASP3 expression levels were significantly elevated by the overexpression and reduced by the knockdown of ATF3 in NPCs, and knockdown of ATF3 also reversed the TBHP-induced NPC phenotype (Figures 3(c)–3(f)). Furthermore, results of ELISA also revealed that silencing ATF3 repressed IL-1*β* secretion induced by TBHP (Figures 3(h) and 3(i)). Additionally, ferroptosis repressor SLC7A11 and antioxidant protein SOD2 were decreased in ATF3-overexpressing NPCs, whereas knockdown of ATF3 contributed to the opposite phenotype (Figures 3(e) and 3(f)). There was almost no change in the expression of these proteins in TBHP-treated ATF3-knockdown NPCs (Figure 3(g)). Collectively, these data uncovered that ATF3 might not only enhance TBHP-induced NPC FAoptosis and ROS production by repressing SLC7A11 and SOD2 but also promote ECM degradation and IL-1*β* secretion.

### 3.4. Silencing ATF3 Alleviated IDD in a Rat Model

**Figure 4:**
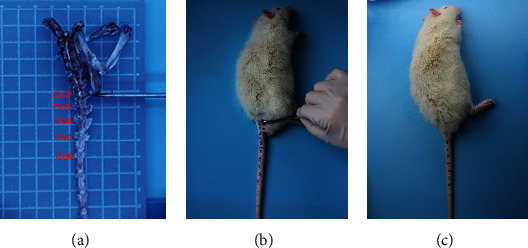
The anatomical characteristics of the intervertebral disc of the rats' tail. (a) The rats' specimens of the pelvis and proximal caudal bone. The scalpel handle indicates that the Co2 transverse process was flush with the lower edge of the ischial tuberosity. (b) The ischial tuberosity and the transverse process of C_O_2-Co4 were regarded as the anatomical landmarks for manual palpation and positioning. (c) Manual palpation was used to locate the intervertebral space and level of the rats' tail.

Considering that the rat tail is cylindrical without distinct anatomical features, it is hard to pinpoint the levels of the IVD except via using X-ray examination. The IVD of the rat tail was punctured at least twice at different times, and the first puncture marks were likely to heal before the second puncture. Thus, we first developed a manual palpation method to pinpoint the IVD levels without using X-ray examination to overcome these problems. The right transverse process of Co2 was flushed with the ischial tuberosity (Figures 4(a) and 4(b)). We also observed that the transverse processes of Co2-Co4 were relatively larger, and that of the Co5 was smaller, while that of Co6 was barely touchable. Co6 could be touched at the junction between the body skin and the tail skin at the root of the rat tail (Figures 4(a)–4(c)). To locate the IVD segments and keep the marker ever-present, we chose an antidiscoloration marker to mark the rat tail. The rat tail was pulled out of the cage for a few seconds to paint it on the original marked site once a week, without anesthesia. To locate the puncture sites, a manual palpation method was performed each time. In addition, the accurate sagittal section could be obtained by truncating Co4/5 of the rat tail and marking with the transverse process of Co5 as a reference.

**Figure 5:**
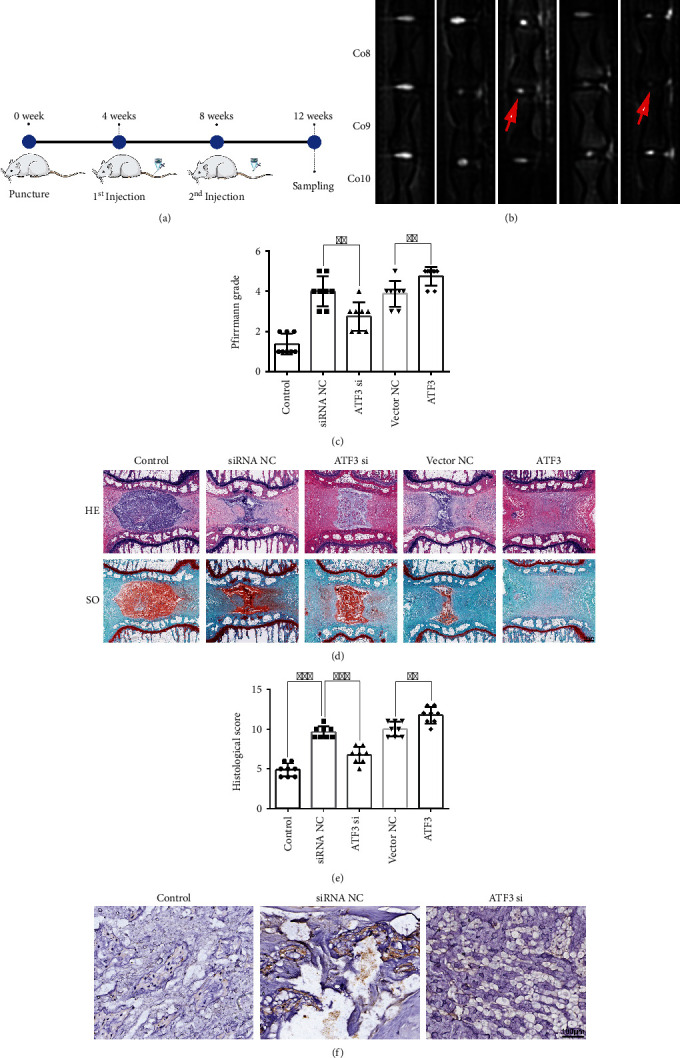
Silencing ATF3 alleviated IDD in a rat model. (a) A flow chart of the experimental procedure with injections of ATF3, ATF3 si, or negative control at 4 and 8 weeks after surgery. (b) MRI was performed in the IVDs of the rat tail at 12 weeks after surgery. Co8/9 was punctured, while Co7/8 and Co9/10 were in perfect condition. (c) The degree of IDD in the rat at 12 weeks after surgery was evaluated using Pfirrmann's classification method based on the analysis of MRI. ^∗∗^*P* < 0.01. (d) HE (top) and safranin-O/fast green (bottom) staining of the rat NP tissues at 12 weeks after surgery. Scale bar = 600 *μ*m. (e) The histological scores of each group were analyzed based on the histological grading scale. ^∗∗^*P* < 0.01 and ^∗∗∗^*P* < 0.001. (f) Immunohistochemical analysis of the expression level of ATF3 in different groups in the rat NP tissues. Scale bar = 100 *μ*m.

Ji et al. [21] demonstrated that the 31 G needle could induce IDD in the rat tail without obvious complications. To establish a model of IDD, we selected a 31 G needle to puncture the Co8/9 and Co7/8 and Co9/10 without surgery as a control (Figure 5(a)). In the 4th and 8th weeks after surgery, adenoviruses containing ATF3 or ATF3 siRNA were injected into the punctured IVDs (Figure 5(a)). MRI examination unveiled that the Co8/9 became “black” in the ATF3 group and “white” in the ATF3 si group as compared to the vector NC and siRNA NC groups, respectively, at 12 weeks after surgery (Figure 5(b)). The average Pfirrmann grade obtained after analyzing the MRI results significantly decreased in the ATF3 si group (Figure 5(c)), suggesting that silencing of ATF3 could alleviate IDD in rats. HE and SO staining unveiled that there was an obvious boundary between NP and AF with the large and round NPC population located in the NP, which contained a mass of ECM components in the control group, whereas the vector NC and siRNA NC group showed the significantly reduced NP volume and cell density (Figure 5(d)). In the ATF3 si group, we also observed a remarkable recovery in NP volume and cell density (Figure 5(d)). Additionally, the boundary between the NP and AF disappeared in the ATF3 group (Figure 5(d)). The results of the histological score further supported the protective role of silencing ATF3 (Figure 5(e)). Immunohistochemical analysis of the expression level of ATF3 was significantly downregulated in the ATF3 si group in the rat NP tissues (Figure 5(f)). These findings suggested that silencing ATF3 could alleviate the progression of IDD.

### 3.5. Predicting miR-874-3p May Be the Key Upstream Regulator of ATF3

**Figure 6:**
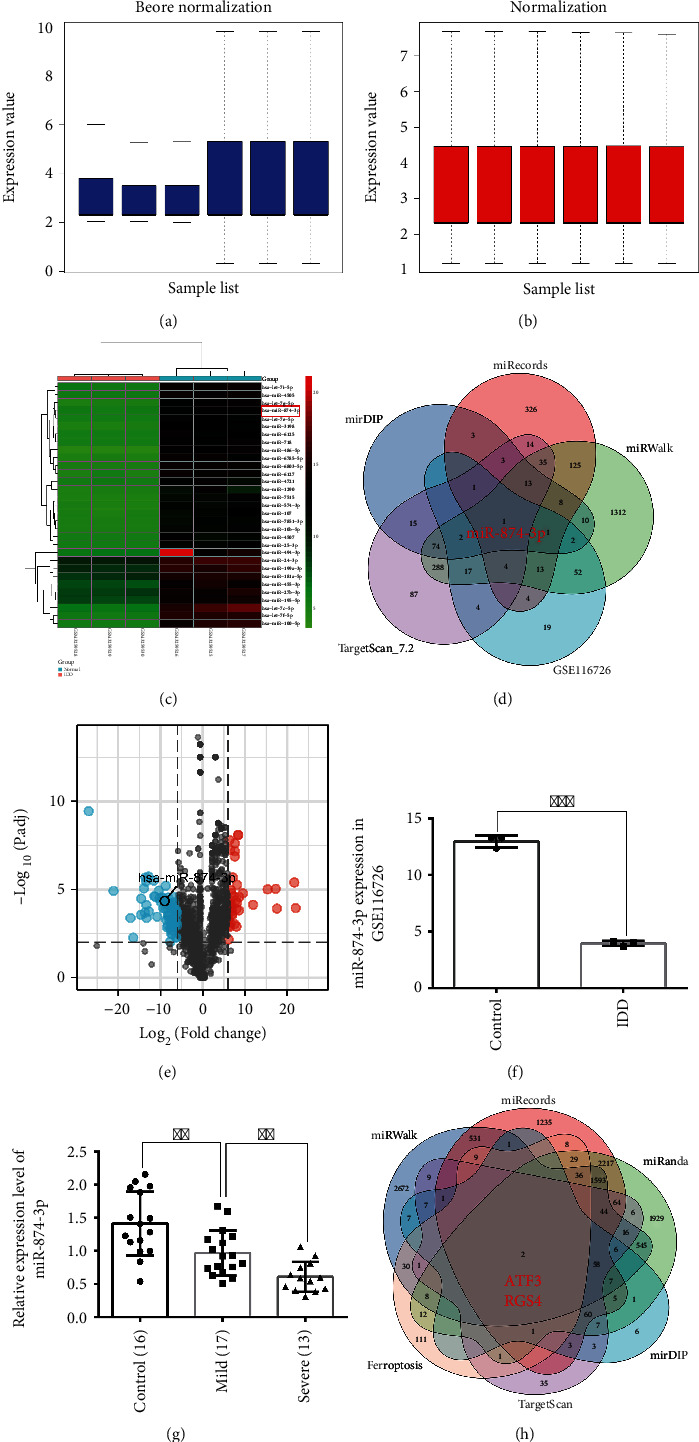
Prediction of miR-874-3p was the upstream regulator of ATF3. (a, b) The box plot unveiled the distribution trends of the 3 normal and 3 degenerative NP samples before and after data normalization. The row indicates the sample, and the column indicates the data expression value in the sample. (c) The heat map shows the downregulated DEMs in the GSE116726 dataset, with rows indicating DEMs and columns indicating the sample. (d) Prediction of the upstream miRNA of ATF3 through the intersection of different databases. (e) The volcano plot shows the 120 DEMs based on the analysis of the GSE116726 dataset. Green points represent downregulated miRNAs (left side) and red points represent upregulated miRNAs (right side). (f) The expression of miR-874-3p in the GSE116726 dataset. ^∗∗∗^*P* < 0.001. (g) The qRT-PCR experiment was used to detect the expression level of miR-874-3p in IDD patients. ^∗∗^*P* < 0.01. (h) Prediction of the downstream target genes of miR-874-3p related to ferroptosis through the intersection of different databases and ferroptosis genes.

The underlying mechanisms by which ATF3 expression is modulated remain largely unclear. We sought to identify the upstream regulators of ATF3. The differentially expressed miRNAs (DEMs) play a crucial role in the initiation and development of IDD by targeting key genes [19–22]. We reanalyzed the IDD-related GSE116726 dataset using the criteria of |log_2_*FC*| > log_2_6 and −log_10_(*adj*.*P* > 2). The box plot (Figures 6(a) and 6(b)) unveiled that the distribution trends of the 3 normal and 3 degenerative NP samples were on the same line after normalization compared with those before normalization. A total of 120 DEMs (77 downregulations) were identified. The heat map revealed that miR-874-3p shows a significant imbalance (Figure 6(c)). miR-874-3p was predicted to be significantly downregulated in the GSE116726 dataset (Figures 6(e) and 6(f)). Moreover, only miR-874-3p was predicted to be the overlapping IDD-related miRNA after merging different databases (Figure 6(d)). Consistent with the trend using the bioinformatics analysis, qRT-PCR demonstrated that the miR-874-3p level was low in mildly degenerative NP tissues and lower in severely degenerative NP tissues, suggesting that the expression of miR-874-3p is negatively correlated with the severity of IDD (Figure 6(g)). Conversely, multiple databases and ferroptosis-related genes downloaded from FerrDb database (Supplementary Table 2 ) were merged to predict the target genes of miR-874-3p, encompassing ATF3 (Figure 6(h)). From the above results, we predicted that miR-874-3p may be the key upstream regulator of ATF3.

### 3.6. The Upregulation of ATF3 in IDD May Be Caused by the Downregulation of miR-874-3p

**Figure 7:**
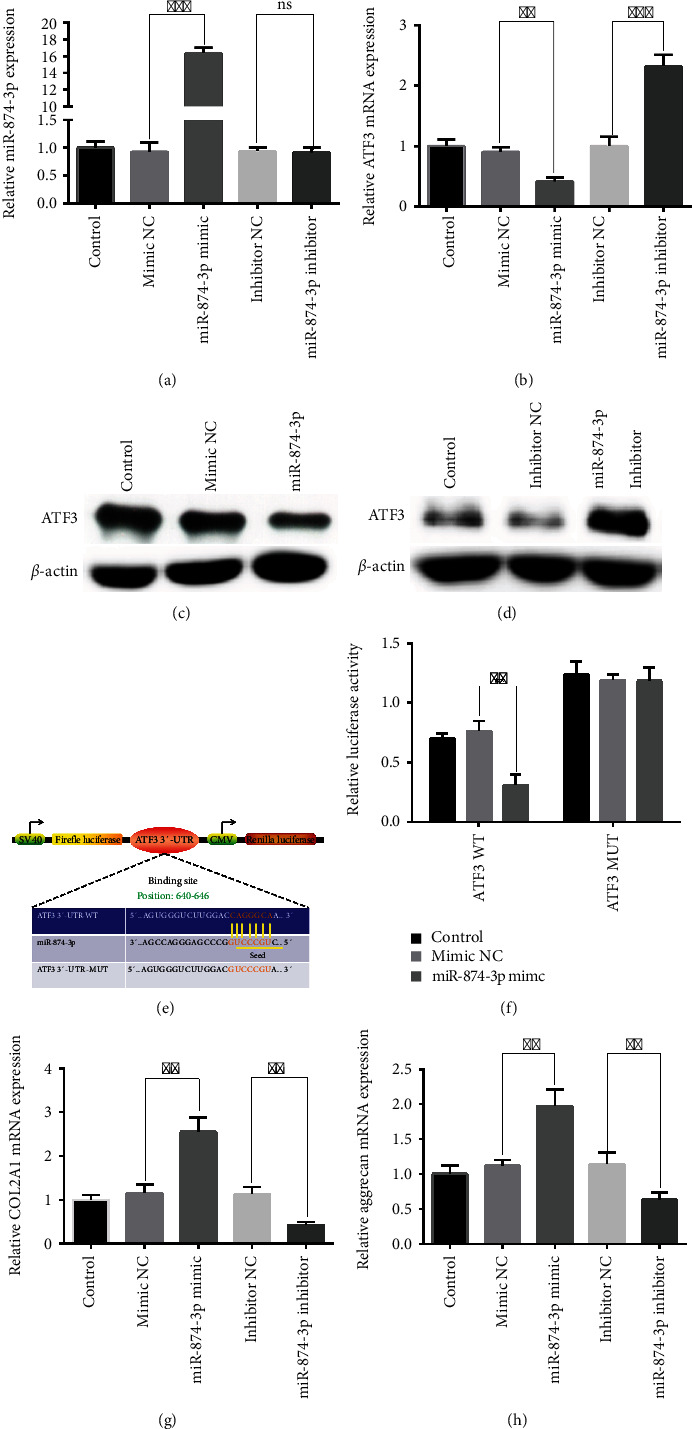
ATF3 was the direct target of miR-874-3p. (a) The expression levels of miR-874-3p were detected in NPCs by qRT-PCR after transfection with miR-874-3p mimic, miR-874-3p inhibitor, or corresponding NC. ^∗∗∗^*P* < 0.001. (b) miR-874-3p negatively regulated the mRNA expression level of ATF3 in NPCs. ^∗∗^*P* < 0.01 and ^∗∗∗^*P* < 0.001. (c) Overexpression of miR-874-3p decreased the protein expression level of ATF3 in NPCs. (d) Knockdown of miR-874-3p increased the protein expression level of ATF3 in NPCs. (e) The TargetScan database showed the putative binding site of ATF3 on miR-874-3p. (f) The dual-luciferase reporter experiment was conducted to measure the activity of wild-type ATF3 or mutant ATF3 after transfection with miR-874-3p mimic or mimic NC into the HEK-293T cell. ^∗∗^*P* < 0.01. (g) miR-874-3p promoted the mRNA expression level of COL2A1 in NPCs. ^∗∗^*P* < 0.01. (h) miR-874-3p promoted the mRNA expression level of Aggrecan in NPCs. ^∗∗^*P* < 0.01.

To confirm whether miR-874-3p is the key upstream regulator of ATF3, a series of experiments were conducted to explore their relationship. miR-874-3p mimic and its inhibitor were transfected into NPCs. The miR-874-3p mimic remarkably elevated the level of miR-874-3p, whereas the expression of miR-874-3p was not altered by the inhibitor (Figure 7(a)), suggesting that the miR-874-3p inhibitor might only competitively bind endogenous miR-874-3p in NPCs, thereby contributing to less endogenous miR-874-3p binding to downstream target genes. Overexpression of miR-874-3p negatively regulated the mRNA and protein expression levels of ATF3, whereas the miR-874-3p inhibitor exerted the opposite effects in NPCs (Figures 7(b)–7(d)). Furthermore, ATF3 has sequences complementary to the miR-874-3p seed region, as predicted using the TargetScan database (Figure 7(e)). The luciferase reporter assay demonstrated that the miR-874-3p mimic significantly inhibited the luciferase activity of ATF3 WT, whereas the luciferase activity of ATF3 MUT did not change significantly (Figure 7(f)), suggesting that ATF3 can directly bind to miR-874-3p through complementary target sites. Furthermore, Song et al. [41] demonstrated that miR-874-3p promoted the protein expression levels of Aggrecan and COL2A1 in NPCs. Our results also revealed that miR-874-3p positively regulated the mRNA expression levels of Aggrecan and COL2A1 in NPCs (Figures 7(g) and 7(h)), suggesting that miR-874-3p might play a protective role in IDD. Findings of this result suggested that the upregulation of ATF3 in IDD may be caused at least in part by the downregulation of miR-874-3p in IDD, thereby relieving the inhibition of ATF3 by miR-874-3p.

## 4. Discussion

ATF3 belongs to the ATF/cyclic AMP-responsive element binding (CREB) protein family that exerts its transcriptional inhibition or activation effect through interaction with other ATF/CREB members or TFs or binding to its target genes' promoters [16]. The abnormal expression and dysfunction of ATF3 could contribute to a series of pathophysiological responses, including cell death, inflammatory response, ECM metabolism disorder, oxidative stress, endoplasmic reticulum stress [16, 42], and even diseases, such as osteoarthritis [43–45], cancer [16], and cardiovascular diseases [42]. In chondrocytes, ATF3 was expressed in the nucleus and cytoplasm [43–45]. Iezaki et al. [43] found that the expression of ATF3 was significantly upregulated in the osteoarthritis cartilage of both mice and humans, and the knockdown of ATF3 in chondrocytes decreased cytokine-induced IL-6 transcription by inhibiting NF-*κ*B signaling to alleviate the development of osteoarthritis. ATF3 was also reported to directly bind to the proximal *MMP13* AP-1 motif and promote cytokine-induced MMP13 expression in human chondrocytes [44]. The relationship between ncRNA and ATF3 has also been studied in chondrocytes. ncRNA RP11-364P22.2 could bind to ATF3 and facilitate its nuclear translocation, thereby promoting chondrocyte apoptosis and ECM degradation [45]. NPCs have similar biological characteristics and functions as chondrocytes. In our study, we observed that ATF3 expression was positively correlated with the severity of IDD. Loss- and gain-of-function assays unveiled that ATF3 enhanced TBHP-induced ECM degradation and IL-1*β* secretion. Based on this, we believed that TBHP treatment might enhance the nuclear translocation of the cytosolic ATF3 protein, further increasing the level of ATF3 in the nucleus, thereby mediating the pathological process of IDD by promoting the regulation of ATF3 and its target and downstream genes. Mechanistically, we speculated that ATF3 may promote IL-1*β* secretion by activating the NF-*κ*B signaling pathway. The increase in ECM degradation may be due to the increased expression of MMPs or IL-1*β* caused by ATF3. However, whether ATF3 directly binds to Aggrecan, COL2A1, or IL-1*β* promoter to regulate their expression needs further studies.

Researchers have long been studying the cell death pathway. Various types of deaths do not exist independently but are closely correlated and interact with each other under pathological conditions [46]. Tang et al. [46] summarized the currently known 12 regulated cell deaths, including apoptosis, pyroptosis, and ferroptosis. Karki and colleagues [47] recently corroborated that the combination of TNF-*α* and IFN-*γ* could induce a mixed cell death consisting of apoptosis, pyroptosis, and necroptosis. Taabazuing et al. [48] uncovered a complex cross-talk between apoptosis and pyroptosis pathways; pyroptotic stimuli can also activate apoptosis through different machineries. We found that ATF3 could promote TBHP-induced NPC apoptosis, ferroptosis, and ROS production via activating c-CASP3 and inhibiting SLC7A11 and SOD2, suggesting ATF3 might mediate the mixed cell death FAoptosis consisting of ferroptosis and apoptosis. The increase in IL-1*β* secretion is a hallmark feature of pyroptosis [49]. Thus, we could not rule out the possibility that the increase in IL-1*β* in NPCs might be attributed to the regulation of pyroptosis of NPCs by ATF3. In addition, whether ATF3 regulates the mixed cell death composed of apoptosis, pyroptosis, and ferroptosis needs to be elucidated.

Another ferroptosis-related TF NFE2L2 was demonstrated to promote ATF3 expression in astrocytes [50]. Brown et al. [51] found that ATF3 was bound to NFE2L2 to inhibit the expression of genes downstream to NFE2L2. Whether ATF3 and NFE2L2 orchestrate to modulate ferroptosis by forming complex feedback loops needs further investigations. Furthermore, endoplasmic reticulum stress has been unveiled to trigger ferroptosis using the unfolded protein response [12]. Thus, future investigations are required to explore whether ATF3 enhances ferroptosis by mediating the endoplasmic reticulum stress and unfolded protein response.

As negative regulators of gene expression, miRNAs play critical roles in the pathological process of IDD [20–22]. Previous investigations revealed the involvement of miR-874-3p in the pathology of the progression of IDD [34, 41]. Additionally, Dai et al. [52] reported that miR-874-3p aggravated renal podocyte injury by regulating apoptosis and oxidative stress. In the current study, we demonstrated that the expression of miR-874-3p significantly decreased in IDD patients. miR-874-3p directly bound to ATF3 to repress the mRNA and protein levels of ATF3. In summary, we speculated that miR-874-3p is significantly downregulated during IDD, thereby relieving the inhibition of ATF3 by miR-874-3p, contributing to a significant increase in ATF3 expression.

The study had the following limitations: Firstly, we collected only 46 clinical samples; more samples are needed to fortify the results. Secondly, the rat intervertebral discs have different biomechanical characteristics than those in humans. Therefore, more animals, such as monkeys or goats, should be included in experimental studies. Thirdly, we did not construct ATF3-knockout mice to further verify the biological effects of ATF3.

## 5. Conclusion

Cumulatively, our results demonstrated that ATF3 was significantly upregulated in IDD patients and TBHP-treated NPCs. The upregulation of ATF3 in IDD may be caused at least in part by the downregulation of miR-874-3p in IDD, thereby relieving the inhibition of ATF3 by miR-874-3p. Under the induction of TBHP, ATF3 might undergo nuclear translocation, further increasing the level of ATF3 in the nucleus, thereby mediating the pathological process of IDD by promoting the regulation of ATF3 and its target and downstream genes. Functionally, ATF3 might not only enhance TBHP-induced NPC FAoptosis and ROS production by repressing SLC7A11 and SOD2 but also promote ECM degradation and IL-1*β* secretion. Furthermore, silencing ATF3 could alleviate IDD in a rat model. Our findings provided novel insights to understand the pathology of IDD, which may reveal novel therapeutic approaches for treating IDD diseases.

## Data Availability

The data used to support the findings of this study are included within the article.

## Supplementary Materials

Supplementary 1 Supplementary Table 1: the detailed data for each patient with different diseases.

Supplementary 2 Supplementary Table 2: the ferroptosis-related genes.
