# Supplementary material for: Silencing ATF3 Might Delay TBHP-Induced Intervertebral Disc Degeneration by Repressing NPC Ferroptosis, Apoptosis, and ECM Degradation
Source: Oxid Med Cell Longev. 2022 Apr 15;2022:4235126. doi: 10.1155/2022/4235126 (PMC9036167; doi:10.1155/2022/4235126)
Supplement: Supplementary 1 — Supplementary Table 1: the detailed data for each patient with different diseases. [file 4235126.f1.docx]

**Supplementary Table 1 The detailed data for each patient with different diseases**

| Number | Gender | Age | Level | Diagnosis | Pfirrmann |
| --- | --- | --- | --- | --- | --- |
| Nor-1 | F | 22 | T12-L1 | Thoracolumbar fracture | I |
| Nor-2 | M | 32 | T12-L1 | Thoracolumbar fracture | II |
| Nor-3 | M | 19 | T12-L1 | Thoracolumbar fracture | II |
| Nor-4 | F | 21 | T12-L2 | Thoracolumbar fracture | I |
| Nor-5 | M | 31 | L1-L2 | Thoracolumbar fracture | II |
| Nor-6 | M | 49 | T12-L1 | Spinal cord injury | II |
| Nor-7 | M | 28 | T12-L1 | Spinal cord injury | I |
| Nor-8 | F | 23 | T12-L1 | Scoliosis | I |
| Nor-9 | F | 12 | L2-L3 | Scoliosis | I |
| Nor-10 | F | 10 | T12-L1 | Scoliosis | I |
| Nor-11 | M | 41 | L4-L5 | Lumbar disc herniation(L5-S1) | II |
| Nor-12 | F | 37 | L4-L5 | Lumbar disc herniation(L5-S1) | II |
| Nor-13 | F | 24 | L5-S1 | Lumbar spondylolisthesis | I |
| Nor-14 | M | 18 | L5-S1 | Lumbar spondylolisthesis | I |
| Nor-15 | M | 29 | L5-S1 | Lumbar spondylolysis | I |
| Nor-16 | M | 32 | L5-S1 | Lumbar spondylolysis | II |
| Mild-1 | M | 57 | L3-L4 | Lumbar disc herniation | IV |
| Mild-2 | M | 57 | L4-L5 | Lumbar disc herniation | IV |
| Mild-3 | F | 31 | L4-L5 | Lumbar disc herniation | III |
| Mild-4 | F | 71 | L3-L4 | Lumbar disc herniation | IV |
| Mild-5 | M | 44 | L4-L5 | Lumbar disc herniation | III |
| Mild-6 | M | 55 | L5-S1 | Lumbar disc herniation | IV |
| Mild-7 | M | 67 | L5-S1 | Lumbar disc herniation | IV |
| Mild-8 | F | 20 | L5-S1 | Lumbar disc herniation | IV |
| Mild-9 | M | 33 | L5-S1 | Lumbar spondylolysis | III |
| Mild-10 | M | 40 | L5-S1 | Lumbar spondylolysis | IV |
| Mild-11 | F | 52 | L4-L5 | Lumbar spondylolysis | IV |
| Mild-12 | M | 64 | L5-S1 | Lumbar spondylolysis | IV |
| Mild-13 | F | 41 | L4-L5 | Lumbar spondylolisthesis | IV |
| Mild-14 | F | 32 | L4-L5 | Lumbar spondylolisthesis | III |
| Mild-15 | M | 65 | L4-L5 | Lumbar spondylolisthesis | IV |
| Mild-16 | M | 45 | L5-S1 | Lumbar spondylolisthesis | IV |
| Mild-17 | F | 31 | L5-S1 | Lumbar spondylolisthesis | III |
| Severe-1 | M | 51 | L4-L5 | Lumbar disc herniation | V |
| Severe-2 | F | 64 | L4-L5 | Lumbar disc herniation | V |
| Severe-3 | F | 70 | L4-L5 | Lumbar disc herniation | V |
| Severe-4 | M | 66 | L5-S1 | Lumbar disc herniation | V |
| Severe-5 | F | 74 | L5-S1 | Lumbar disc herniation | V |
| Severe-6 | M | 76 | L4-L5 | Lumbar disc herniation | V |
| Severe-7 | F | 63 | L4-L5 | Lumbar spondylolisthesis | V |
| Severe-8 | M | 54 | L4-L5 | Lumbar spondylolisthesis | V |
| Severe-9 | M | 57 | L5-S1 | Lumbar spondylolisthesis | V |
| Severe-10 | F | 64 | L5-S1 | Lumbar spondylolisthesis | V |
| Severe-11 | F | 60 | L4-L5 | Lumbar spinal stenosis | V |
| Severe-12 | F | 72 | L5-S1 | Lumbar spinal stenosis | V |
| Severe-13 | M | 68 | L5-S1 | Lumbar spinal stenosis | V |

Abbreviations: Nor, Normal; Mild, Mild degeneration; Severe, Severe degeneration; F, Female; M, Male
