# Supplementary material for: Silencing ATF3 Might Delay TBHP-Induced Intervertebral Disc Degeneration by Repressing NPC Ferroptosis, Apoptosis, and ECM Degradation
Source: Oxid Med Cell Longev. 2022 Apr 15;2022:4235126. doi: 10.1155/2022/4235126 (PMC9036167; doi:10.1155/2022/4235126)
Supplement: Supplementary 2 — Supplementary Table 2: the ferroptosis-related genes. [file 4235126.f2.pdf]

Ferroptosis Genes
ALOX12
ALOX15
ALOX5
ATF3
ATF4
ATP5MC3
CBS
CHAC1
ELAVL1
FTH1
GPX4
HIF1A
HMGB1
HMOX1
HSPB1
IREB2
MAPK14
RPL8
NFE2L2
SLC2A14
SLC3A2
SLC40A1
SLC7A11
TF
TFRC
TP53
VDAC2
LONP1
SRC
STAT3
TMBIM4
TP63
ZFP36
SQSTM1
PROM2
RB1
SCD
SESN2
NFS1
NQO1
OTUB1
PLIN2
PML
PRDX6
HELLS
HSF1

MT1G
MTOR
MUC1
NF2
HSPA5
ISCU
JUN
LAMP2
FTMT
GCH1
GCLC
CHMP6
CISD1
CISD2
ENPP2
FADS2
FANCD2
Fer1HCH
FH
BRD4
CA9
CAV1
AGPAT3
CD44
CDKN1A
CHMP5
AIFM2
AKR1C1
AKR1C2
AKR1C3
ARNTL
ACSL3
ALB
ANGPTL7
ARRDC3
ASNS
ATP6V1G2
AURKA
BLOC1S5-TXNDC
BNIP3
CAPG
CEBPG
CXCL2
DDIT3
DDIT4
DRD4
DRD5

DUSP1
EIF2AK4
EIF2S1
FTL
GABPB1
GDF15
GLUT13
GPT2
GPX2
HAMP
HBA1
HERPUD1
HIC1
HNF4A
HSD17B11
IL33
IL6
JDP2
KIM-1
KLHL24
LURAP1L
MAFG
MAP3K5
MT3
NCF2
NGB
NNMT
NOS2
OXSR1
PCK2
PLIN4
PRDX1
PSAT1
PTGS2
RELA
RGS4
RIPK1
RRM2
SETD1B
SELENOS
SLC1A4
SLC2A1
SLC2A12
SLC2A3
SLC2A6
SLC2A8
SLC7A5

SNORA16A
SP1
SRXN1
STEAP3
STMN1
TFAP2C
TRIB3
TSC22D3
TUBE1
TXNIP
TXNRD1
UBC
VEGFA
VLDLR
XBP1
YWHAE
ZFP69B
ZNF419
CS
EMC2
NOX1
CYBB
NOX3
NOX4
NOX5
DUOX1
DUOX2
G6PD
PGD
PIK3CA
FLT3
SCP2
ACSL4
LPCAT3
NRAS
KRAS
HRAS
TFR2
SLC38A1
SLC1A5
GLS2
GOT1
CARS1
KEAP1
ATG5
ATG7
NCOA4

ALOX12B
ALOX15B
ALOXE3
PHKG2
ACO1
G6PDX
ULK1
ATG3
ATG4D
BECN1
MAP1LC3A
GABARAPL2
GABARAPL1
ATG16L1
WIPI1
WIPI2
SNX4
ATG13
ULK2
SAT1
EGFR
MAPK3
MAPK1
BID
ZEB1
DPP4
CDKN2A
PEBP1
SOCS1
CDO1
MYB
MAPK8
MAPK9
LINC00472
PRKAA2
PRKAA1
BAP1
ABCC1
MIR6852
ACVR1B
TGFBR1
EPAS1
HILPDA
IFNG
ANO6
LPIN1
TNFAIP3

TLR4
ATM
YY1AP1
EGLN2
MIOX
TAZ
MTDH
IDH1
SIRT1
FBXW7
PANX1
DNAJB6
BACH1
ACSF2
